# Experimental Evolution of *Mycobacterium tuberculosis* in Human Macrophages Results in Low-Frequency Mutations Not Associated with Selective Advantage

**DOI:** 10.1371/journal.pone.0167989

**Published:** 2016-12-13

**Authors:** Valentina Guerrini, Selvakumar Subbian, Pierre Santucci, Stéphane Canaan, Maria Laura Gennaro, Gianni Pozzi

**Affiliations:** 1 Public Health Research Institute, New Jersey Medical School, Rutgers, The State University of New Jersey, Newark, New Jersey, United States of America; 2 Laboratory of Molecular Microbiology and Biotechnology, Department of Medical Biotechnologies, University of Siena, Siena, Italy; 3 Aix-Marseille Univ, Centre National de la Recherche Scientifique, Laboratoire d'Enzymologie Interfaciale et de Physiologie de la Lipolyse, Marseille, France; Fundació Institut d’Investigació en Ciències de la Salut Germans Trias i Pujol, Universitat Autònoma de Barcelona, SPAIN

## Abstract

Isolates of the human pathogen *Mycobacterium tuberculosis* recovered from clinical samples exhibit genetic heterogeneity. Such variation may result from the stressful environment encountered by the pathogen inside the macrophage, which is the host cell tubercle bacilli parasitize. To study the evolution of the *M*. *tuberculosis* genome during growth inside macrophages, we developed a model of intracellular culture in which bacteria were serially passaged in macrophage-like THP-1 cells for about 80 bacterial generations. Genome sequencing of single bacterial colonies isolated before and after the infection cycles revealed that *M*. *tuberculosis* developed mutations at a rate of about 5.7 × 10^−9^ / bp/ generation, consistent with mutation rates calculated during *in vivo* infection. Analysis of mutant growth in macrophages and in mice showed that the mutations identified after the cyclic infection conferred no advantage to the mutants relative to wild-type. Furthermore, activity testing of the recombinant protein harboring one of these mutations showed that the presence of the mutation did not affect the enzymatic activity. The serial infection protocol developed in this work to study *M*. *tuberculosis* genome microevolution can be applied to exposure to stressors to determine their effect on genome remodeling during intra-macrophage growth.

## Introduction

Tuberculosis remains a global health concern; the evolution of drug resistance represents a major impediment to anti-tuberculosis efforts. While *Mycobacterium tuberculosis* was long thought to be genetically monomorphic, recent studies have shown that heterogeneity can be found within the infecting bacterial population [1–4]. Indeed, many low-frequency genetic variants, most of which are transient, coexist in clinical samples [5]. *M*. *tuberculosis* genetic heterogeneity observed in samples collected from infected humans at multiple times post-infection [6, 7], in different clinical stages of disease [8], from different anatomical sites [9], or even within the same lesion [8], prompts the question of whether mutations developed during infection confer a bacterial phenotype more adapted to the host environment.

Since *M*. *tuberculosis* resides within host macrophages for much of the infection [10, 11], these phagocytes may represent a potent contributor to *M*. *tuberculosis* genetic heterogeneity. The role of macrophages as driving force for bacterial genome evolution has been suggested by macrophage infection experiments performed with other pathogens [12]. In addition, heterogeneity among intracellular mycobacteria has been observed in short-term macrophage infections *in vitro* [13]. Many factors in the macrophage environment may have a mutagenic effect [14]. For example, oxidative stress has been proposed as a major mutagenic agent encountered by *M*. *tuberculosis* during *in vivo* infection [8]. The macrophage environment may not only induce the development of new mutations, but also operate a selective pressure on *M*. *tuberculosis*, leading to selection and fixation of adaptive mutations in the bacterial population. Typical protocols of macrophage infection are not amenable to long-term bacterial genome evolution studies due to the short lifespan of in *vitro* infected cells.

To determine whether mutations occur in the *M*. *tuberculosis* DNA during macrophage infection, we developed a new model of serial macrophage infections. Tubercle bacilli were passaged in macrophages for multiple, consecutive cycles for a total of 80 bacterial generations; passaging was associated with bacterial mutations that were identified by whole genome sequencing.

## Material and Methods

### Bacterial strains and growth conditions

To establish the initial inoculum for the *M*. *tuberculosis* intracellular culture model, a single colony-derived bacterial culture of *M*. *tuberculosis* H37_Rv_Siena strain (P007027.1) [15] was obtained by disruption of clumps by shaking with glass beads [16] followed by plating on solid medium. A single colony was subcultured to prepare bacterial stocks that were used for the first cycle of THP-1 infection.

*M*. *tuberculosis* cultures were grown in Middlebrook 7H9 broth (Becton Dickinson, Sparks, MD) (liquid medium) or 7H10 (solid medium) (Difco, Franklin Lakes, NJ) supplemented with 0.05% Tween 80, 0.2% glycerol and 10% ADN (2% glucose, 5% bovine serum albumin, 0.15 M NaCl). Liquid cultures of *M*. *tuberculosis* were grown in 25-ml tubes at 37°C with magnetic-bar stirring at 450 rpm and optical density was measured at 580 nm with a densitometer. Plates were incubated at 37°C in sealed plastic bags.

### Macrophage culture and differentiation

The THP-1 human monocytic cell line was obtained from the American Type Culture Collection (ATCC TIB-202). Cells were grown in RPMI 1640 medium containing 2 mM L-glutamine (Corning Cellgro, Manassas, VA) and supplemented with 10% fetal bovine serum (FBS) (Sigma-Aldrich, St. Louis, MO), 100 U/ml penicillin and 100 μg/ml streptomycin (Sigma-Aldrich, St. Louis, MO), maintaining a concentration of 1 × 10^5^ to 5 × 10^5^ cells/ml. Cell aliquots were frozen in FBS containing 10% dimethylsulfoxide. For each infection experiment, one aliquot was thawed and cells were grown for four passages and subsequently differentiated by treatment with 40 nM phorbol 12-myristate 13-acetate (PMA) (Sigma-Aldrich, St. Louis, MO) for 72 h at the concentration of 5 × 10^5^ cells/ml in 25 cm^2^ cell culture flasks (Costar, New York, NY). Cell differentiation following PMA stimulation was monitored over time by microscopic observation of cell morphology, plastic adherence proprieties, and analysis of CD11b and CD11c macrophage marker expression [17, 18].

To analyze marker expression, 1 × 10^6^ cells were stained with Cy-Chrome-conjugated anti-human CD11b and PE-conjugated anti-human Cd11c (BD Biosciences, San Jose, CA) for 30 min at 4°C, washed, resuspended in 1 × PBS, and analyzed by flow cytometry with a FACSCalibur instrument (BD Biosciences, San Jose, CA). The number of total and viable adherent cells was determined in all experiments by detaching cells from the flasks, staining with 0.4% Trypan Blue solution (Sigma-Aldrich, St. Louis, MO) and counting in a TC10 automated cell counter (Biorad, Hercules, CA).

### Serial passaging of *M*. *tuberculosis* in THP-1 cells and collection of single-colony isolates

Infections were performed as previously described [16]. For the first cycle of infection, an aliquot of the bacterial culture was defrosted and diluted in RPMI 1640 medium containing 2mM L-glutamine and 10% FBS. After clump disaggregation by shaking with 3mm-diameter glass beads, one aliquot of the bacterial culture was serially diluted and plated for CFU determination and a second aliquot was added to four 25 cm^2^ tissue culture flasks (2.1 × 10^4^ CFU/flask), each containing 2.1 × 10^6^ THP-1 cells to obtain an expected multiplicity of infection (MOI) of 1:100 (bacterial CFU:cells). After one day of incubation at 37°C in 5% CO_2_ atmosphere, medium was removed, and cell monolayers were washed with 1× PBS for three times to remove extracellular bacteria. One flask was used for THP-1 count while a second flask was used to determine the number of intracellular bacterial CFU. In this second flask, THP-1 cells were lysed by adding 0.01% sodium dodecyl sulphate (SDS) in ice-cold water and incubating at room temperature for 20 min. The cell lysate was centrifuged at 3,800 × *g* for 30 min and the sedimented bacteria were resuspended in liquid medium for CFU enumeration. Fresh medium was added to the remaining two flasks that were incubated for six more days. At this time, cell culture medium was removed and the cell monolayers were washed with 1 × PBS three times. One flask was used to check the viability of THP-1 cells. Cells from the second flask were lysed as described above to release intracellular bacteria for the next infection cycle. Bacteria were collected by centrifugation of cell lysates, resuspended in RPMI medium, and treated with glass beads (as described above) to infect a new THP-1 culture. An aliquot of this suspension was used for CFU count. Following this procedure, ten serial infection cycles were performed. The number of bacterial intracellular generations was calculated for each cycle by using the following formula: (log_2_ CFU day 7- log_2_ CFU 18 h)/log_2_ 2.

Thirteen single colonies were collected from the bacterial inoculum and from the intracellular bacterial population harvested at the end of the ten infection cycles for genomic DNA extraction and whole genome Illumina sequencing.

### DNA genomic extraction

Genomic DNA was extracted from heat-inactivated bacteria by using the cetyltrimethylammonium bromide (CTAB)-lysozyme protocol, essentially as previously described [19]. To increase the efficiency of mycobacterial disruption, mechanical lysis was performed utilizing 150–212 μm-diameter acid washed glass beads (Sigma-Aldrich, St. Louis, MO) and a Vortex Genie 2 disrupter (Stratech Scientific ltd, Oaks Drive, UK).

### Illumina sequencing and identification of genetic variations

Whole genome sequencing was performed at IGA Technology Services S.r.l. (Udine, Italy) using Illumina 2 × 100 bp paired-end reads HiSeq 2000 system. Reads were trimmed, quality filtered, and mapped against H_37_RvSiena as a reference sequence (CP007027.1) using the Mosaik Assembler software [20]. Genome fraction coverage and read depth were analyzed by Qualimap software [21].

Single nucleotide substitutions, insertions, and deletions were retrieved with the VarScan software [22] by setting a minimum read depth of fifteen and a minimum variant frequency of 50% required to call a variant. This cutoff was selected to exclude variants located in genomic regions containing prophages and repetitive sequences, where most sequencing errors occur. The mutations identified were validated by PCR amplification and Sanger sequencing (Sanger sequencing was performed by Eurofins MWG Operon, Ebersberg, Germany). The primers used for amplification and sequencing are listed in S1 Table.

Large genomic fragment mutations relative to the sequence of the H_37_RvSiena reference strain were evaluated as follows: the filtered reads obtained from the sequencing of each genome were *de novo* assembled by using Ray 2.3.0 [23] and the alignment of the contigs to H_37_RvSiena genome sequence was visualized by using Mauve 2.3.1 [24]. For polymorphisms located in coding sequences of genes, variations in amino acid sequences were predicted using the translation Expasy tool (http://web.expasy.org/translate/). Presence and location of a signal peptide and conserved protein domains were evaluated by TatP 1.0 (http://cbs.dtu.dk/services/TatP/), SignalP 4.1 (http://cbs.dtu.dk/services/SignalP/), and Pfam (http://pfam.sanger.ac.uk/) searches.

### Mutation frequency and rate

To determine the frequency of the identified mutations in bacterial populations, aliquots of the inoculum and the tenth cycle culture were subjected to glass bead treatment and plated on agar plates for single-colony isolation. 37 single-colony isolates were collected and bacteria were mechanically lysed by shaking with 150–212 μm acid washed glass beads (Sigma-Aldrich, St. Louis, MO) in Tissue Lyser instrument (Qiagen, Hilden, Germany). Beads and unbroken bacteria were sedimented by centrifugation, and supernatant was used as PCR template. Amplification and Sanger sequencing of the regions containing the mutations were performed using the primers indicated in S1 Table. *M*. *tuberculosis* mutation rate was estimated by using a standard formula [8].

### THP-1 infection with *M*. *tuberculosis cut3* Val223Ala and *plcA* His39Arg mutant isolates

THP-1 cells were differentiated with 40 nM PMA for 3 days in 24 well plates (Costar, New York, NY) at a concentration of 5 × 10^5^ cells per well. For infection, aliquots of wild type, *cut3* Val223Ala and *plcA* His39Arg mutant bacteria were defrosted and diluted in RPMI 1640 medium containing 2 mM L-glutamine and 10% FBS to obtain an MOI of 1:10 (bacterial CFU:cells). After clump disruption by glass bead treatment, the bacterial culture was added to THP-1 cells. After one day of infection, the cell monolayers were washed three times with 1 × PBS to remove extracellular bacteria. Fresh medium was added to the culture and incubation was continued for six additional days. Bacterial CFU and THP-1 viability were determined at days 1, 3 and 7. For these purposes, the adherent cells were detached by incubation with 5 mM EDTA in 1 × PBS for 20 minutes, counted, and lysed with 0.05% SDS. The lysates were serially diluted and plated on agar plates for CFU enumeration. The CFU counts were normalized to the number of adherent THP-1 cells. Each experiment was performed in triplicate.

### Murine model of infection

Eight-week-old female BALB/c mice were infected with approximately 300 CFU via the aerosol route with wild type, *cut3* Val223Ala, and *plcA* His39Arg mutant strains. At 3h (time zero) and 2, 4, 8 and 12 weeks post-infection, 3–4 mice per condition were sacrificed by cervical dislocation. For bacterial CFU enumeration, one lung per mouse was removed aseptically and homogenized, and serial dilutions of organ homogenates were plated on solid medium. At 12 weeks post-infection, one lung per mouse infected with wild type and *cut3* Val223Ala mutant was fixed in neutral formalin and embedded in paraffin for hematoxylin and eosin, and Ziehl-Neelsen staining. Lung sections were evaluated in a blinded manner for semi-quantitative evaluation of pathology, which was based on the following scoring criteria: 0- intact lung (no granuloma); 1- increased cellularity; 2- granulomas occupying up to 25% of lung section; 3- granulomas occupying 25–50% of lung section; 4- granulomas occupying 50–75% of lung section, and 5- granulomas occupying >75% of lung section with necrosis. The morphometric analysis of involved lung area and granuloma size was performed using Sigmascan Pro Software (Systat Software Inc, San Jose, CA), as described [25].

### Cloning, expression, and purification of the wild type and His39Arg mutant PlcA protein

Purified genomic DNA of *M*. *tuberculosis* H_37_RvSiena wild type and *plcA* His39Arg mutant strains was used as template for PCR amplification of *plcA* gene by using the primers shown in S2 Table. The PCR products were digested with BsphI and HindIII restriction enzymes, purified, and cloned into the pMyC plasmid at the Nco1 and HindIII cloning sites. The gene was under the control of the acetamidase promoter, as previously described [26]. Recombinant plasmid pMyC-*plcA* was analyzed by nucleotide sequencing (GATC biotech) and used to transform *Mycobacterium smegmatis* mc^2^ 155 GroEL1ΔC [27]. Expression and purification of recombinant wild type and His39Arg mutant PlcA proteins were performed as described by Bakala N’ Goma *et al*. [26]. Briefly, transformed *M*. *smegmatis* mc^2^ 155 GroEL1ΔC was grown in Middlebrook 7H9 medium supplemented with 10% ADN, and 50 μg/ml Hygromycin B at 37°C with shaking to OD_600_ of 3. Expression of recombinant proteins was induced by treatment with 0.2% acetamide for 16 h. After induction, the cells were sedimented by centrifugation and resuspended in 30 ml of ice cold buffer A (10 mM Tris/HCl pH 8.0, 150 mM NaCl) prior to mechanical lysis with a French Press device at 1100 psi. After centrifugation of the lysate at 17,000 × *g*, the pellet was resuspended in ice cold buffer A, sonicated as described previously [26], and incubated with 1% N-laurylsarcosine at 4°C for 16 h. After incubation, samples were centrifuged at 17,000 × *g* for 30 min and then at 27,000 × *g* for 20 min at 4°C. Supernatants were loaded onto a ProBond Ni^2+^-agarose column (Thermo Fisher Scientific, Waltham, MA) pre-equilibrated in buffer A supplemented with 0.5% N-laurylsarcosine. The column was washed with 60 ml of buffer A, and elution was performed with a 20 mM to 500 mM imidazole gradient. Recombinant proteins eluted at 100 mM and 250 mM imidazole were analyzed by SDS-PAGE electrophoresis onto a 12% polyacrylamide gel. Fractions were pooled, dialyzed overnight at 4°C against buffer A, concentrated to obtain a final concentration of approximately 1 mg/ml, and stored at -80°C.

### Phospholipase C assay

Phospholipase C activity of the recombinant wild type and mutant PlcA protein was tested by using the Amplex Red Phosphatidylcholine-Specific PLC Assay Kit and a spectrofluorimeter (Thermo Fisher Scientific, Waltham, MA), according to the manufacturer’s instructions. *Bacillus cereus* phospholipase C and heat-inactivated recombinant PlcA were used as positive and negative controls, respectively. The enzymatic activity (EA) corresponds to the mole number of fluorescent fatty acid release from the phosphatidylcholine per minute in arbitrary unit (AU). Specific activity was expressed in pmol/min and per mg of protein.

### Statistical analysis

Two tailed Student’s *t*-test was used to analyze the data; statistical significance was defined as *P* < 0.05.

### Nucleotide sequencing data availability

The full genome sequence of the strain *M*. *tuberculosis* H_37_RvSiena is available from the NCBI GenBank database, with the accession number CP007027.1.

### Ethics statement

The animal study was carried out in strict accordance with the recommendations in the Guide for the Care and Use of Laboratory Animals of the National Institutes of Health. The protocol was approved by the Institutional Animal Care and Use Committee of Rutgers, the State University of New Jersey (permit number: 14020D0417).

## Results and Discussion

### A model of extended intracellular growth of *M*. *tuberculosis*

**Fig 1:**
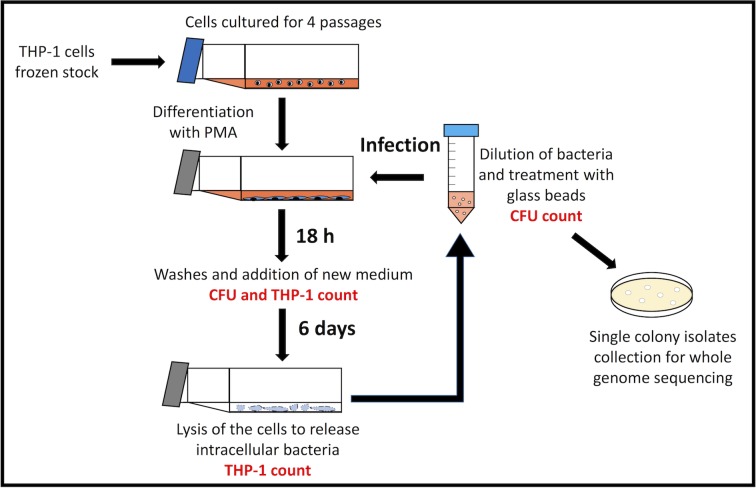
Schematic representation of the long-term intracellular *M*. *tuberculosis* culture protocol. For each infection cycle, a frozen stock of THP-1 cells was thawed and cells were cultured for 4 passages. Cells were differentiated by PMA stimulation and infected with *M*. *tuberculosis*. After 18 h of infection, medium was replaced, and infected cells were incubated for 6 days. At day 7, THP-1 cells were lysed to release the intracellular bacteria, which were diluted in cell culture medium and subjected to clump disaggregation as described in Methods. Approximately 1/10 to 1/20 of this bacterial suspension was used to infect a new THP-1 cell culture. A total of 10 serial infection cycles were performed, and bacterial CFU and THP-1 cells were counted at 18 h and 7 days of infection for each cycle. An aliquot of the initial bacterial inoculum and of the intracellular bacteria collected at the end of the tenth cycle were diluted and spread onto agar plates for single-colony isolation and whole genome sequencing.

To obtain long-term intracellular growth of *M*. *tuberculosis*, we developed a protocol in which THP-1 cells were serially infected with *M*. *tuberculosis* for a total of 10 infection cycles (Fig 1). Infection was performed with fully differentiated macrophages, as demonstrated by microscopic analysis, plastic adherence proprieties, and expression of macrophage markers (S1 and S2 Figs).

**Fig 2:**
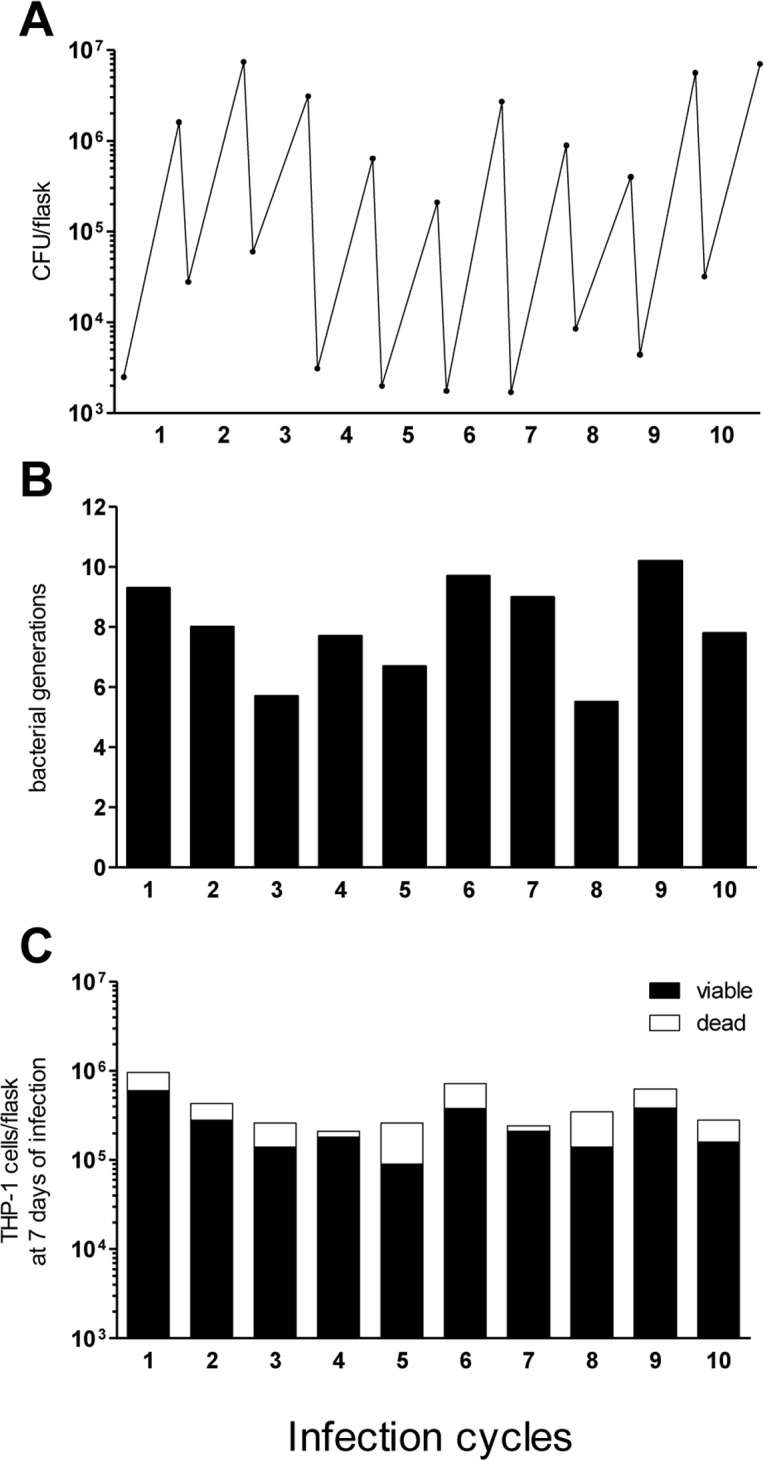
Parameters of the continuous intracellular culture model. THP-1 cells were infected with *M*. *tuberculosis* H37_Rv_Siena strain for 10 serial infection cycles. The graphs show the parameters evaluated for each cycle. **(A) Bacterial CFU count.** Bacterial CFU at day 1 and day 7 of infection. **(B) Intracellular generations.** Number of bacterial generations per cycle, calculated by using the formula (log_2_ CFU day 7- log_2_ CFU day 1)/log_2_ 2. **(C) Count of infected THP-1 cells.** Number of total, viable adherent THP-1 cells (black bar), and dead cells (white bar) at day 7 of infection.

Multiple parameters were evaluated in each cycle. These included bacterial CFU counts at 1 and 7 days of infection, number of bacterial generations, and number of total and viable adherent THP-1 cells (Fig 2). The bacterial growth rate did not change significantly across cycles (the slight variation observed in CFU at day 1 of infection can be presumably attributed to the variability of the MOI) (Fig 2A). The number of bacterial generations per cycle showed a moderate fluctuation, ranging from 5 to 10 (Fig 2B), for a total of 79.6 generations. Moreover, the proportion of viable cells in the cultures was relatively constant across cycles, with averages of ~80% and 60% viable cells at 1 and 7 days of infection, respectively (Fig 2C). Overall, no significant change was observed in host cell viability and bacterial growth across cycles.

### Identification and frequency of mutations

To assess the presence of genetic variants among isolates in the bacterial population, whole genome sequencing was performed for selected single-colony isolates obtained from the inoculum and from the tenth cycle of infection (Table 1). This comparison was necessary to distinguish mutations existing prior to infection from those that may have been acquired during infection. An average read depth of 325× and a mean genome coverage of 99.86% was obtained. Low (< 10×) read depth was obtained only for genomic regions containing prophages or highly repeated sequences such as Y980_0336 (13E12 repeat family gene), Y980_1575 (PhiRv1 prophage element), Y980_2933 (*ppsC*), Y980_0746 (*pe_pgrs9*), Y980_1450c (*pe_pgrs27*), Y980_3508 (*pe_pgrs54*) and Y980_3478 (*ppe60*) genes, as also observed in previous studies [28].

The sequencing results showed that 9 out of 13 single-colony isolates obtained from the inoculum were identical to the H37_Rv_Siena reference strain (wild type isolates). In addition, four thymine to cytosine mutations were found (Table 1): (i) **T**-1216143-**C** in the 3’ non coding region of *celA2b* (Y980_1090) [29] was present in three isolates (ii) **T**-4284317-**C** (Thr237Ala) in the coding region of *papA2* (Y980_3820c) was present in one isolate. Sequencing of 13 single-colony isolates from the tenth cycle of intracellular growth yielded 10 isolates having the wild-type genotype and three isolates presenting a single-point mutation. All mutations were thymine to cytosine transitions: (i) **T-**1216143-**C** in the 3’ non coding region of *celA2b* (Y980_1090), as found in the inoculum, (ii) **T-**2631348-**C** (His39Arg) in the coding region of *plcA* gene (Y980_2351c), and (iii) **T-**3872665-**C** (Val223Ala) in the coding region of *cut3* gene (Y980_3451) (Table 1). The latter two mutations were not found in the inoculum.

**Table 1:** Mutations identified in *M*. *tuberculosis* genome and their frequency.

			Frequency^*b*^	
Mutation^*a*^	Gene (position or amino acid substitution)	Annotation/ Comments	Inoculum	10^th^ cycle
T-1216143-C	*celA2b* (3’ non coding region)	Putative cellulase/ Hydrolyzes barley β-glucan [30]	9/50 (18%)	5/50 (10%)
T-4284317-C	*papA2* (Thr237Ala)	Polyketide synthase associated protein/ Involved in sulfolipid-I synthesis [31, 32]	1/50 (2%)	0/50 (0%)
T-2631348-C	*plcA* (His39Arg)	Phospholipase C 1/ Hydrolyzes macrophage phospholipids [26] Translocated by Tat system [33]	0/50 (0%)	1/50 (2%)
T-3872665-C	*cut3* (Val223Ala)	Cutinase precursor/ Primary trehalose dimycolate hydrolase in *M*. *tuberculosis* [34]	0/50 (0%)	1/50 (2%)

^*a*^ All four mutations are thymine to cytosine transitions. The number between the nucleotides indicates the position of the mutation in the genome of *M*. *tuberculosis* H_37_RvSiena (CP007027.1).

^*b*^ Number of single-colony isolates bearing the mutation relative to the total number of the isolates sequenced, and relative percent (in parenthesis).

To determine the frequency of the four T→C transitions, an additional 37 single-colony isolates from the bacterial inoculum and from the tenth infection cycle were subjected to targeted re-sequencing (S1 Table). The transition **T**-1216143-**C** (3’ non coding *celA2b*) was present in the inoculum and at the tenth cycle at a similar frequency (18% and 10%, respectively). The mutations **T**-4284317-**C** (*papA2*), **T**-2631348-**C** (*plcA*) and **T**-3872665-**C** (*cut3*) were not found in additional isolates, indicating a very low frequency occurrence (≤ 2%). If we assume that **T**-2631348-**C** (*plcA*) and **T**-3872665-**C** (*cut3*) were acquired during intracellular growth, the estimated mutation rate was ≥ 5.7 × 10^−9^ / bp/ generation (since prophages and repetitive sequences were excluded from the analysis due to high probability of sequencing errors, mutations that may have been present in those regions are not included in this calculation). This number is in accordance with the predicted *M*. *tuberculosis in vivo* mutation rate [8].

### *In vitro* and *in vivo* growth of *cut3* Val223Ala and *plcA* His39Arg mutants

**Fig 3:**
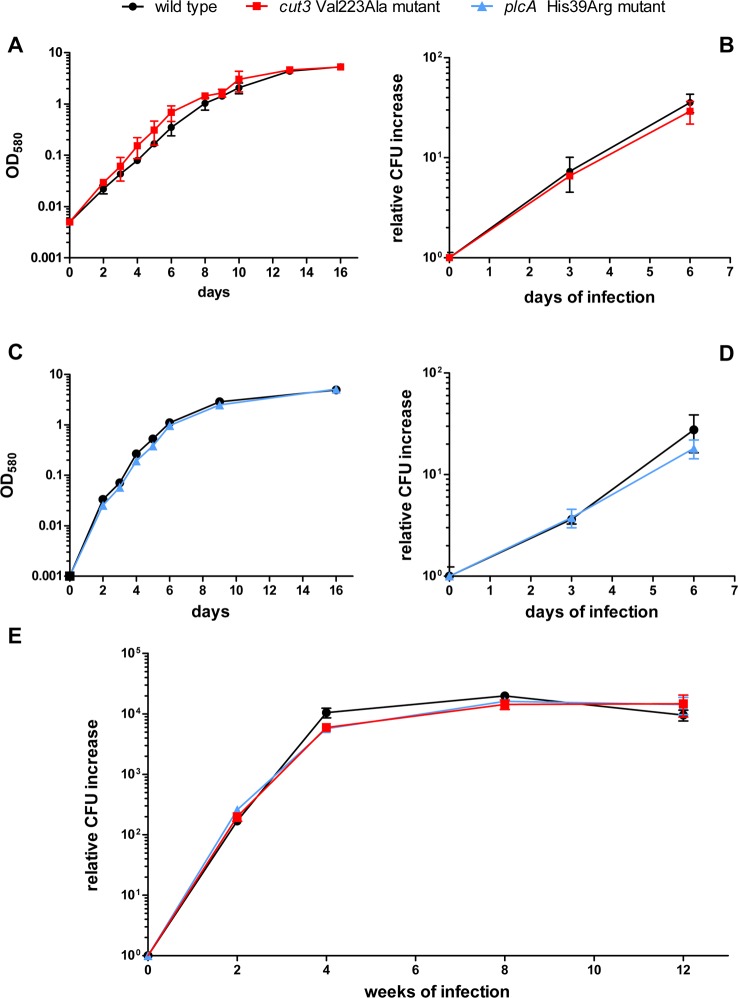
Growth curves of wild type, *cut3* Val223Ala and *plcA* His39Arg mutant strains in liquid medium, THP-1 cells and murine lung. **(A and C) Growth curves in liquid medium**. Growth curves in liquid medium over 16 days of incubation for *cut3* Val223Ala mutant (**A**) and *plcA* His39Arg mutant (**C**) strains. **(B and D) Growth curves in THP-1 cells**. Growth curves of *cut3* Val223Ala mutant **(B)** and *plcA* His39Arg mutant (**D**) strains in THP-1 cells over 6 days of infection. Data are expressed as CFU increase relative to day 0. **(E) Growth curves in murine lung.** Growth curves of wild type, *cut3* Val223Ala mutant, and *plcA* His39Arg mutant strains in the murine lung up to 12 weeks post-infection. Panels A-D show the mean and standard deviation of results obtained from three replicates; panel E shows the mean and standard deviation of results obtained from 3–4 animals per time point.

We asked whether the mutations identified at the tenth infection cycle but absent from the inoculum were associated with a fitness advantage. To test this possibility, we compared growth rates of mutant and wild type bacteria in axenic cultures and in infected THP-1 cells. No difference was observed (Fig 3A–3D). Moreover, determination of lung bacillary load at different time points after mouse infection showed comparable growth rates for mutant and wild type bacteria (Fig 3E). Thus, no differences were observed in the growth of both *cut3* Val223Ala and *plcA* His39Arg mutants relative to wild type cells *in vitro* or during infection.

### Histopathological analysis of murine lungs infected with wild type and *cut3* Val223Ala mutant

**Fig 4:**
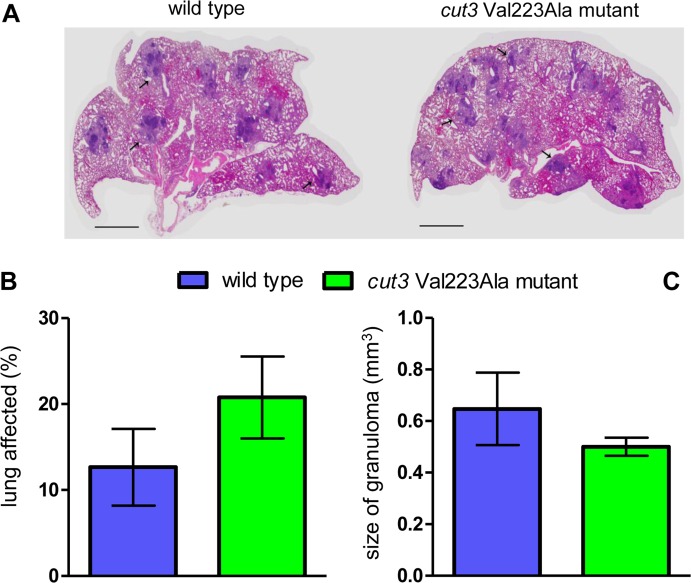
Morphometric analysis of mouse lungs infected with wild type and *cut3* Val223Ala mutant strains at 12 weeks of infection. **(A) Representative image of mouse lung sections.** The image represents 4× magnification of lung sections stained with hematoxylin and eosin. Arrows indicate granulomatous lesions. Scale = 1mm. **(B-C) Proportion of involved lung and granuloma size.** The graphs show the proportion of lung parenchyma occupied by granulomas **(B)** and the size of granulomas **(C)**. Results represent the mean and standard deviation of data collected from 3 mice per infecting bacterial strain.

Gross inspection of lungs harvested at 12 weeks of infection from mice infected with wild type and *cut3* Val223Ala mutant strains, revealed multiple, well-formed granulomas, characteristic of chronic *M*. *tuberculosis* infection (Fig 4A). No difference was observed between lung tissue infected with mutant and wild type bacteria in terms of proportion of lung parenchyma occupied by granulomas (involved lung) (Fig 4B) or size of lung granulomas (Fig 4C) (*P* > 0.05).

Accordingly, the disease score for these two strains were also similar (2.67+/-0.57 for the *cut3* Val223Ala mutant strain and 2.33+/-0.57 for the wild type strain) (*P* > 0.05). Thus, infection of murine lungs with wild type and mutant bacteria induced similar histopathological effects.

### Biochemical characterization of wild type and His39Arg mutant PlcA recombinant protein

**Fig 5:**
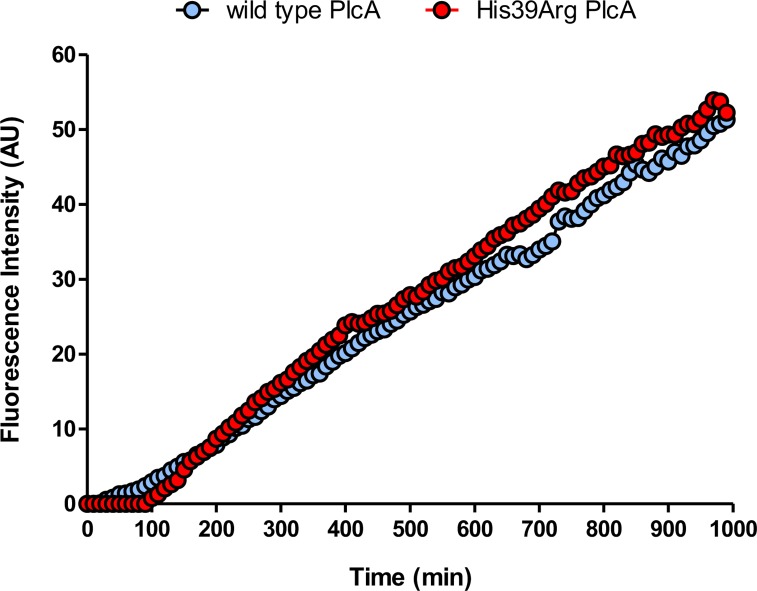
Phospholipase C activity testing. Time-course hydrolysis of phosphatidylcholine (PC) with 100μg of wild type (blue circles) and His39Arg mutant (red circles) recombinant PlcA. The release of phosphocholine was measured indirectly by the fluorescence measurement of resorufin (AU) released using the Amplex red phosphatidylcholine kit, and continuously monitored at λ_exc_ of 510 nm and λ_em_ of 590 nm. Blue and red curves represent the mean of three independent experiments (coefficient of variation was 0.0195 and 0.0293, respectively).

In order to identify the effect of the *plcA* mutation on enzymatic activity, wild type and His39Arg mutant PlcA enzymes were purified from a recombinant *M*. *smegmatis* strain, and their activities were compared *in vitro*. No difference in rate of phosphatidylcholine hydrolysis was observed between the two enzymes: the enzymatic activity was 50.8 and 55.2 mAU/min and the specific activity was 16.32 and 17.74 pmol/min/mg for the wild type and mutant protein, respectively (Fig 5). Therefore, the presence of the His39Arg mutation had no effect on the enzymatic activity of PlcA.

## Conclusions

In conclusion, whole genome sequencing of *M*. *tuberculosis* grown in macrophages for ~80 generations showed genetic heterogeneity in the bacterial population that is not associated with fitness advantage. Since genetic variants of *M*. *tuberculosis* are also found *in vivo* at a frequency similar to that obtained in our experiments [1–4], our results suggest that most of the mycobacterial diversity arising during infection is likely associated with non-adaptive mutations. Our study does not exclude the possibility that phenotype-changing mutations of *M*. *tuberculosis* occur during infection. In our protocol, the frequency of such mutations may have been below the limit of detection of our study. Alternatively, the macrophage model used in this study might have imposed insufficient stress to *M*. *tuberculosis* to select for adaptive mutations. Refinements of the model will have to include use of activated macrophages to determine whether the frequency of adaptive mutations of *M*. *tuberculosis* increases when macrophages express higher antibacterial activity. The long-term macrophage infection system developed in the present study makes it possible to investigate bacterium-macrophage interactions over a time span that is much longer than conventional macrophage infections. Moreover, addition of drugs at various concentrations might make our cyclic infection protocol useful for the study of development of drug resistance.

## Supporting Information

S1 Fig Morphology and adhesion propriety of THP-1 cells upon PMA stimulation. THP-1 cells were incubated with 40nM PMA for 72 h. At 24 h, 48 h and 72 h of PMA incubation, cell morphology was observed under a light microscope at a magnification of 60 × **(A-B-C)**. At the same time points, culture medium was discarded, and three washes performed to remove non-adherent cells. Adherent cells were gently detached from the flask by using a cell scraper, and the number of viable cells was determined. The results are expressed as percentage of viable adherent cells relative to the number of initially stimulated cells **(D)**. Each point represents the mean ± standard deviation of three replicates.
(PDF)

S2 Fig Time course of surface marker expression in THP-1 stimulated with PMA. THP-1 cells were stimulated with 40nM PMA for 72 h. At 24 h (purple line), 48 h (green line) and 72 h (blue line) of PMA treatment, cells were stained with fluorophore-conjugated anti-human CD11b **(B)** and anti-human CD11c **(D)** antibodies, and analyzed by flow cytometry. Unstimulated cells were analyzed for CD11b **(A)** and CD11c **(C)** expression as controls. Cells not incubated with antibodies were included in each experiment (pink line).
(PDF)

S1 Table Oligonucleotides used in PCR and Sanger sequencing. (PDF)

S2 Table Oligonucleotides and restriction enzymes used to obtain PlcA recombinant proteins. (PDF)
