# Supplementary figures and images for: Experimental Evolution of Mycobacterium tuberculosis in Human Macrophages Results in Low-Frequency Mutations Not Associated with Selective Advantage
Source: PLoS One. 2016 Dec 13;11(12):e0167989. doi: 10.1371/journal.pone.0167989 (PMC5154527; doi:10.1371/journal.pone.0167989)

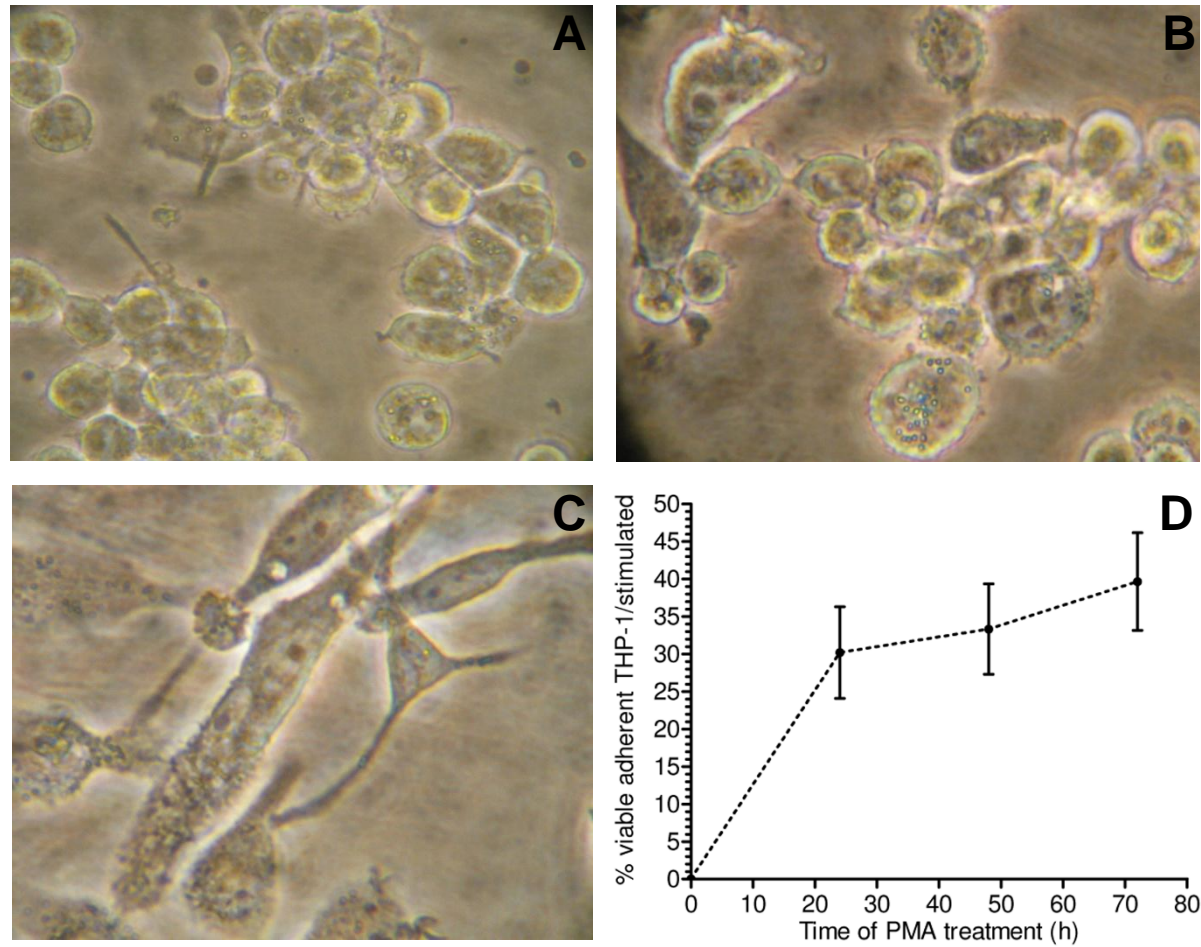

**S1 Fig. Morphology and adhesion propriety of THP-1 cells upon PMA stimulation.**

Supplement: S1 Fig — THP-1 cells were incubated with 40nM PMA for 72 h. At 24 h, 48 h and 72 h of PMA incubation, cell morphology was observed under a light microscope at a magnification of 60 × (A-B-C). At the same time points, culture medium was discarded, and three washes performed to remove non-adherent cells. Adherent cells were gently detached from the flask by using a cell scraper, and the number of viable cells was determined. The results are expressed as percentage of viable adherent cells relative to the number of initially stimulated cells (D). Each point represents the mean ± standard deviation of three replicates. (PDF) [file pone.0167989.s001.pdf]

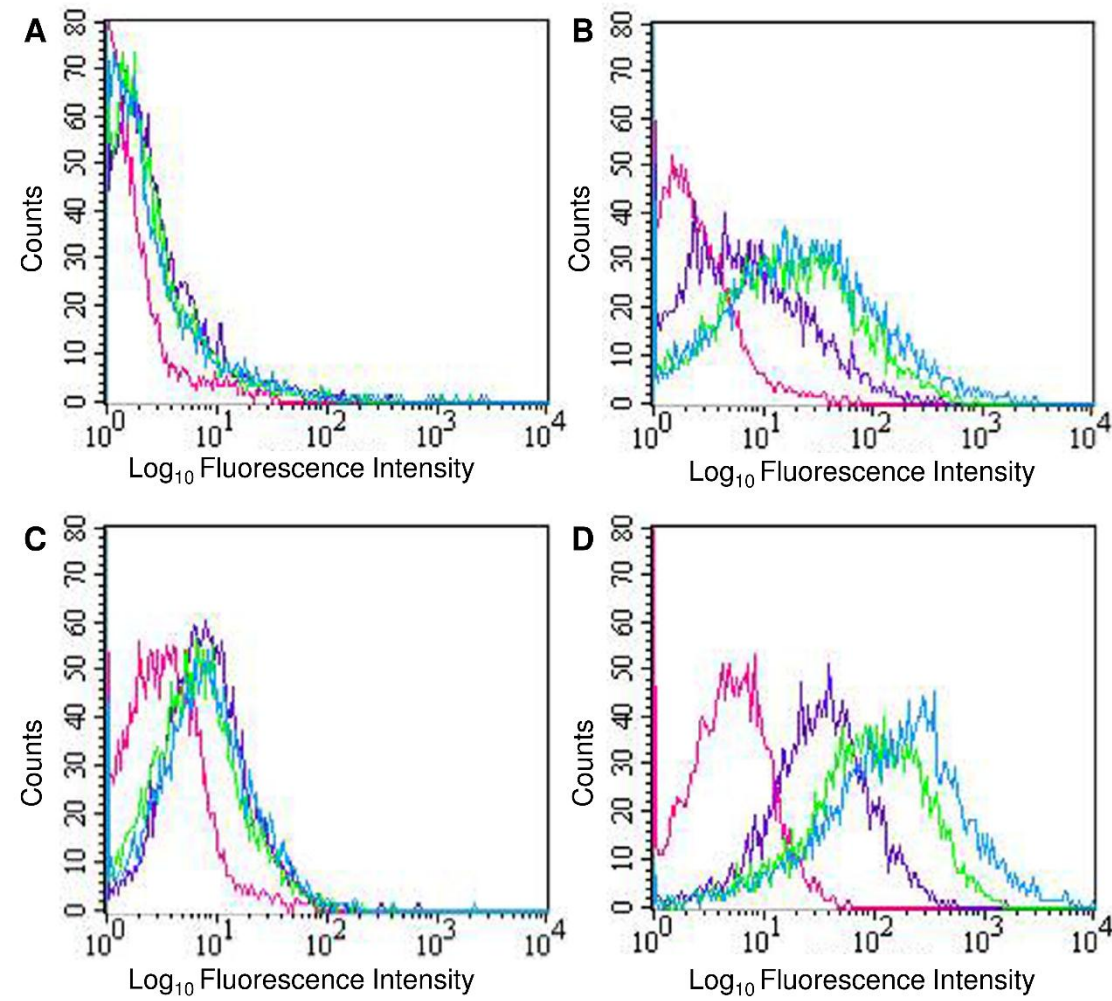

**S2 Fig. Time course of surface marker expression in THP-1 stimulated with PMA.**

Supplement: S2 Fig — THP-1 cells were stimulated with 40nM PMA for 72 h. At 24 h (purple line), 48 h (green line) and 72 h (blue line) of PMA treatment, cells were stained with fluorophore-conjugated anti-human CD11b (B) and anti-human CD11c (D) antibodies, and analyzed by flow cytometry. Unstimulated cells were analyzed for CD11b (A) and CD11c (C) expression as controls. Cells not incubated with antibodies were included in each experiment (pink line). (PDF) [file pone.0167989.s002.pdf]
