# Supplementary material for: Experimental Evolution of Mycobacterium tuberculosis in Human Macrophages Results in Low-Frequency Mutations Not Associated with Selective Advantage
Source: PLoS One. 2016 Dec 13;11(12):e0167989. doi: 10.1371/journal.pone.0167989 (PMC5154527; doi:10.1371/journal.pone.0167989)
Supplement: S1 Table — (PDF) [file pone.0167989.s003.pdf]

S1 Table. Oligonucleotides used in PCR and Sanger sequencing

Target	Primer name	Oligonucleotide sequence (5’-3’)
celA2b (3’ non coding)	Rv41	GTTCTCGGCGAAAGTCAACTA
	Rv42	ACCCGATGTTCAAGCAAGCG
plcA	Rv47	CGGAATGTCACGTCGAGAGTTT
	Rv48	CCCATTGGTGCTGGAAAGCGTT
papA2	Rv49	ACGAGCAACAGGGCGAGCGATT
	Rv50	CGTTCTGAGTTCTGTCGGCGTCCTA
cut3	Rv55	GCGGACACATCGACGG
	Rv56	GCATCTGAAGGACAGACC
