# Supplementary material for: Experimental Evolution of Mycobacterium tuberculosis in Human Macrophages Results in Low-Frequency Mutations Not Associated with Selective Advantage
Source: PLoS One. 2016 Dec 13;11(12):e0167989. doi: 10.1371/journal.pone.0167989 (PMC5154527; doi:10.1371/journal.pone.0167989)
Supplement: S2 Table — (PDF) [file pone.0167989.s004.pdf]

S2 Table. Oligonucleotides and restriction enzymes used to obtain PlcA recombinant proteins

Oligonucleotide sequence (5'-3')[a]	Restriction enzyme
CATGGCGTCATGAACTCACGTCGAGAGTTTTTGACAAAGC	BspHI
CATGGCGAAGCTTGGCTGCACAGCCCGCTGGG	HindIII

[a] Restriction site sequences are underlined
